# Hepatitis B and hepatitis C prevalence among people living with HIV/AIDS in China: a systematic review and Meta-analysis

**DOI:** 10.1186/s12985-020-01404-z

**Published:** 2020-08-24

**Authors:** Songxia Yu, Chengbo Yu, Jian Li, Shiming Liu, Haowen Wang, Min Deng

**Affiliations:** 1 Zhejiang Provincial Key Laboratory for Drug Clinical Research and Evaluation, The First Affiliated Hospital, College of Medicine, Zhejiang University, Hangzhou, 310003 China; 2 State Key Laboratory for Diagnosis and Treatment of Infectious Diseases, Collaborative Innovation Center for Diagnosis and Treatment of Infectious Diseases, The First Affiliated Hospital, College of Medicine, Zhejiang University, Hangzhou, 310003 China; 3 Department of Interventional medicine, Zhejiang Provincial People’s Hospital, Hangzhou, 310003 Zhejiang China

**Keywords:** Hepatitis B, Hepatitis C, HIV, Meta-analysis, Coinfection

## Abstract

**Background:**

There has been little published data on estimates of HBV and/or HCV coinfection in HIV-positive patients in China or an understanding of how this coinfection varies with different factors. Therefore, this study aimed to determine, through a systematic review and meta-analysis, the prevalence of HBV and/or HCV in HIV-positive patients in China and explore variations in prevalence.

**Methods:**

The Medicine, Web of Science, Chinese Web of Knowledge, and Wanfang databases were searched using a search strategy combining key words and related disease-specific subject terms to identify relevant cohort or cross-sectional studies published up to April 2019. Included articles were assessed for quality. Pooled prevalence and 95% confidence intervals (CIs) were calculated according to study region and other specific characteristics.

**Results:**

Our searches identified 7843 records, but only 66 studies were included in our meta-analysis. The pooled HBsAg prevalence in HIV-positive patients was 13.7% (95% CI 12.3–15.3%), with variations found in terms of age and geographic region. The meta-HCV prevalence was 24.7% (95% CI 19.3–30.5%), which varied over the study period and age. The pooled HBV-HCV coinfection prevalence was 3.5% (95% CI 2.4–4.8%), with variations found in terms of age and geographic region.

**Conclusion:**

Given the high burden of HBV and HCV coinfections in HIV-positive patients, the incorporation of comprehensive screening, treatment, prevention, and vaccination programs into general HIV management in China is imperative.

## Background

The launch and advancement of antiretroviral therapy (ART) has significantly decreased the mortality rates among people living with HIV/AIDS (PLWHA) worldwide and transformed HIV/AIDS from a lethal disease to a chronic manageable condition [1]. Although China has a low HIV/AIDS epidemic in the general population, the number of PLWHA continues to increase. The Chinese Center for Disease Control and Prevention (CDC) estimated that there were approximately 500,000 people who were infected with HIV by the end of 2014 [2], and at the end of June 2017, this number had changed to 660,000, including 41.7% of whom were already AIDS patients [3]. China is also a country with a heavy disease burden for hepatitis B (HBV) and hepatitis C (HCV). Globally, there were an estimated 2 billion people who were infected with HBV, one-third of whom resided in China [4, 5]. China is also one of the countries with the greatest number of chronic HCV infections, with an estimate of 9.8 million chronic HCV infections [6].

Since HIV, HBV and HCV share similar transmission routes, coinfections are very common in PLWHA and are associated with long-term morbidity and mortality; therefore, the consequences of coinfections exceed the impact of any single virus alone [7]. HIV infection can accelerate the course of hepatitis B and hepatitis C, manifesting a faster progression to fibrosis and cirrhosis [8, 9]. Similarly, liver disease is one of the most important non-AIDS causes of death in PLWHA [10–12], especially in the era of ART. In addition, the treatment of HIV in patients with HBV and/or HCV coinfection is difficult because ART may increase the risk of hepatotoxicity. Therefore, there is concern that HBV and/or HCV may threaten the success of ART programs in developing countries. It is essential to estimate the prevalence and disease characteristics of HBV and/or HCV coinfection in PLWHA.

HIV, HBV, and HCV share similar routes of transmission, which are predominantly through intravenous drug use, transfusion of blood or blood products, and sexual contact. However, there is no national-level study to estimate the prevalence of HBV and HCV in PLWHA in China. Previous studies have only been conducted at a single site or focused on specific populations, so those studies failed to provide a comprehensive profile of hepatitis virus–HIV coinfection in China [13–15]. A few multicenter studies in China indicated that the HBsAg seroprevalence in PLWHA ranged from 8.7 to 12.5%, while the seroprevalence of anti-HCV ranged from 12.2 to 41.8% [16–18]. While those studies may be informative in the local geographic sites, they may not entirely represent the situation in China as a whole. Therefore, this study aimed to summarize the available information to evaluate the prevalence of HBV and HCV in PLWHA in China. The findings of this study will be used to guide future intervention strategies and enhance the repertoire of actions for both preventative and clinical purposes.

## Main text

### Search strategies

The review was conducted in accordance with the PRISMA (Preferred Reporting Items for Systematic Reviews and Meta-Analyses) Statement [19]. We conducted a comprehensive literature search in the Medicine, Web of Science, Chinese Web of Knowledge, and Wanfang databases. In each literature database, we cross-referenced MeSH terms of “HIV” and “Hepatitis B” or “Hepatitis C”. We also searched using the following key words: “HIV”, “HBV”, “HCV”, “coinfection”, and “co-morbidity”. Moreover, to supplement these database searches, hand searches of reference lists of included articles were also performed. All identified articles were downloaded, stored, managed, and reviewed with EndNote (version X8).

### Inclusion and exclusion of studies

In this review, we only included studies that reported chronic HBV infection prevalence rate or chronic HCV infection rate among PLHWA in China or contained enough data (e.g., number of HBV/HCV-positive cases and sample size) to compute those prevalence rates in PLHWA. Our study only focused on laboratory diagnosis of chronic HBV infection or HCV infection; thus, we did not include studies in which HBV and HCV prevalence were estimated with self-reported data or review of medical records. We included prospective or retrospective cohort, cross-sectional, case-control, and experimental studies. We excluded publications with possible severe selection bias (e.g., estimation of HBV prevalence among hepatocellular carcinoma patients). Studies that reported HBV or HCV prevalence in fewer than 50 patients were excluded. Our review only targeted studies published in English or Chinese. For duplicate studies, we only retained the version with the most complete dataset and excluded the others.

### Data extraction and quality assessment

Two authors (SX.Y. and J.L.) screened titles and abstracts to remove clearly ineligible studies. Two authors (SX.Y. and J.L.) then independently performed a full-text review of the remaining articles for eligibility, with discrepancies resolved by consensus. For eligible studies, the following information was extracted and stored: the name of the first author, year of publication, year (period) of study conducted, region of China, type of study design, demographics of participants (i.e., age and gender), number of PLWHA subjects involved (sample size), and number of HBV- or HCV-positive subjects. The extracted data can be found in Supplementary Table 1.

We assessed the quality of all included studies using an adapted version of bias tool for prevalence studies, which was developed by Munn and colleagues [20]. We used a scoring system to assess the quality of the included studies as commonly implemented in other prevalence meta-analyses [21]. Studies were awarded one point for one of four items covering probability sampling, described description of the study subjects and the settings, adequate sample sizes (≥200 people), and high response rates for laboratory tests of HBV and HCV (> 90%). A total of four points could be assigned to each individual study. We then classified studies with a total score of three or four points to be of high quality, whereas two points represented moderate quality and scores of one or zero suggested low quality.

### Statistical analysis

Using the R meta package, we calculated the pooled prevalence of HBV, HCV and HBV-HCV and 95% confidence intervals (CI), as well as I^2^ values and Q statistic *p*-values, to assess study heterogeneity [22]. To stabilize variances, we transformed individual study prevalence estimates using the Freeman-Tukey double arcsine transformation [23, 24]. We used random-effects models [25] because we expected substantial heterogeneity among studies. Therefore, each prevalence estimate should be interpreted as an average prevalence across studies with true differences in target population prevalence, not a common prevalence across studies with the same target population prevalence [26]. In addition, we conducted a sensitivity analysis to investigate the influence of a single study on the pooled prevalence estimate by omitting each study in each turn. Publication bias was investigated using Egger’s test and Peter’s test, and if statistically significant bias was found, the trim and fill method was employed to adjust for possible publication bias.

We also conducted a series of subgroup analyses to investigate the potential sources of heterogeneity. The following factors were considered in the subgroup analysis: The study period (comparison of studies conducted after 2010 with those before 2010), geographical region (comparison of 6 regions of China), study design, mean age (comparison of ≤40 years old with > 40 years old), proportion of males (comparison of studies with more than ≥80% of males with fewer than that), and study quality (comparison of studies with quality score ≥ 3 with those with fewer than 3).

## Results

### Included studies search process

**Fig. 1:**
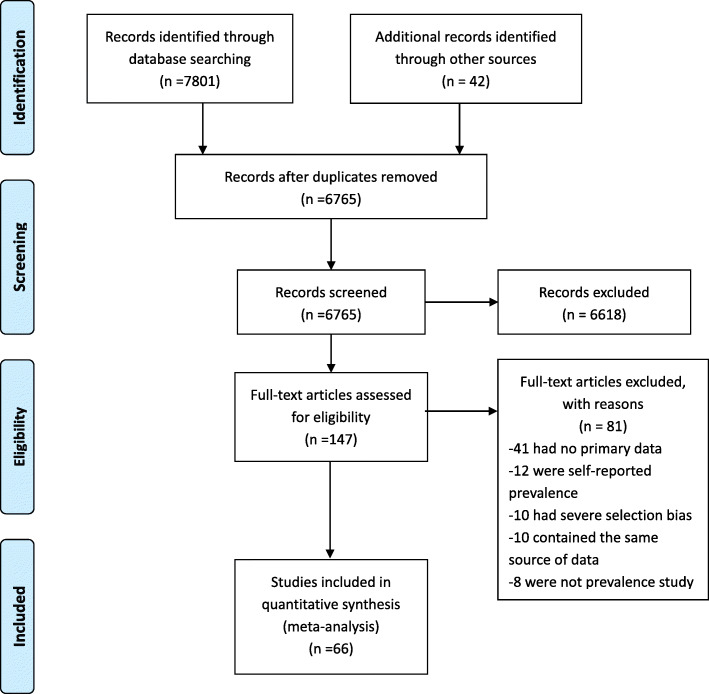
PRISMA Flow Diagram

Our searches initially identified a total of 7843 records. After removal of duplicates, we reviewed the titles of 4765 papers. Based on the title screening, a total of 245 papers were eligible for abstract screening, which further reduced the number of papers requiring full-text review to 147. A total of 66 papers that finally met the inclusion criteria and contained or allowed the estimation of coinfection of HBV or HCV in HIV-infected patients were selected for inclusion in this review. Figure 1 illustrates the PRISMA flow diagram.

### Characteristics of included studies

The characteristics of the included studies are summarized in Supplementary Table 1. Of the 66 studies included in this review, 46 studies reported the HBV prevalence among PLWHA, 59 estimated the HCV prevalence among PLWHA, and 30 had HBV-HCV infections among PLWHA. Study breakdown from the six regions of China were as follows: East China 14, North China 9; Central China 14, South China 9, Northwest China 5, and Southwest China 8. In addition, 7 studies were conducted across multiple regions. Most of the studies (53 studies) were retrospectively designed, and 13 were cross-sectional studies. Most of the studies (37 studies) were hospital-based, and 26 were community-based. More than half (42 studies) were published before 2010, and 18 studies were published after 2010. There are only eight studies published in English, and the others were published in Chinese. A total of 42 studies were deemed high-quality studies among all included studies.

### Meta-analysis of HBsAg prevalence in PLWHA

**Fig. 2:**
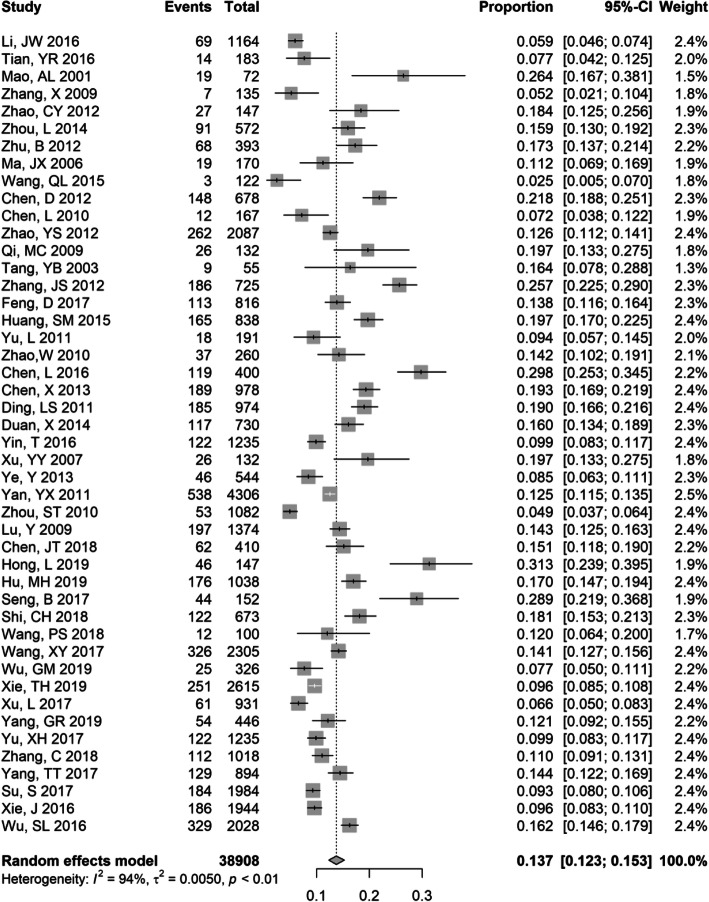
Forest plot showing meta-analysis of HBsAg prevalence in HIV-positive populations using random effects model. The vertical dotted line indicates the pooled prevalence of all studies combined

The HBsAg prevalence estimates (Supplementary Table 1) for the included studies ranged from the highest of 31.3% (95% CI: 23.9–39.5%) to the lowest of 2.5% (95% CI: 0.5–7.0%) among PLWHA. There is a total of 46 studies reporting HBsAg prevalence among 38,908 PLWHA. The pooled HBsAg prevalence, using the Der Simonian-Laird random-effects model, was 13.7% (95% CI 12.3–15.3%) among PLWHA (Fig. 2), and the sensitivity analyses omitting one study at a time yielded consistent results, with a narrow range from 13 to 14% (Supplementary Fig. 1). The heterogeneity was substantial among the estimates (I^2^ = 94%, *p* = 0.005) (Fig. 2). There was no substantial variation in the subgroup analysis, including study period, study design, proportion of gender, and study quality (Table 1). However, the prevalence of HBsAg was higher in studies with a mean age above 40 (16.2, 95% CI: 14.1–18.4%) than in studies with a mean age ≤ 40 (12.3, 95% CI: 10.3–14.4%). There were also geographic variations in pooled HBsAg prevalence. Generally, lower HBsAg prevalence rates were observed in Northwest China (10.2, 95% CI: 9.2–11.2%) and Southwest China (13.1, 95% CI: 10.6–15.9%) than in other regions of China, including North China (13.1, 95% CI: 8.1–19.2%), East China (14.3, 95% CI, 11.2–17.6%), South China (16.7, 95% CI, 13.1–20.6%), and Central China (15.4, 95% CI, 11.4–19.8%).

**Table 1:** Meta-analysis of Prevalence in Sub-groups

Variable	HBV		HCV		HBV-HCV	
	Prevalence,%	95%CI	Prevalence,%	95%CI	Prevalence,%	95%CI
Study Period						
Before 2010	13.4	(11.6,15.4)	31.7	(24.8,39.2)	3.6	(2.3,5.3)
2010 ~ 2019	14.7	(11.5,18.1)	8.5	(5.4,12.3)	3.5	(0.9,7.7)
Study design						
Retrospective	13.6	(11.8,15.5)	23.4	(17.8,29.5)	5.0	(3.2,7.2)
Cross-sectional	14.2	(11.9,16.6)	30.2	(16.2,46.4)	3.3	(2,4.8)
Gender						
Male (≥80%)	13	(10.8,15.4)	21	(10.4,34)	4.0	(2.2,6.4)
Others	13.9	(11.9,16)	24.9	(18.6,31.8)	3.1	(1.6,5.1)
Study Quality						
High (Score ≥ 3)	13.5	(11.8,15.3)	21.3	(15.1,28.3)	3.6	(2.1,5.4)
Low	14.4	(10.6,18.6)	30.8	(20.6,42)	3.3	(1.7,5.4)
Mean Age						
≤ 40	12.3	(10.3,14.4)	31.1	(21.9,41.1)	4.1	(2.4,6.3)
> 40	16.2	(14.1,18.4)	12.2	(3.5,25.2)	1.5	(0.7,2.6)
Geographic region						
North China	13.1	(8.1,19.2)	28.8	(12.0,49.5)	3.0	(2.1,4.1)
East China	14.3	(11.2,17.6)	19.5	(10.1,31.0)	1.9	(0.7,3.7)
South China	16.7	(13.1,20.6)	26.3	(11.1,45.0)	4.8	(1.0,11.0)
Central China	15.4	(11.4,19.8)	21.3	(11.5,33.1)	3.9	(1.0,8.5)
Northwest China	10.2	(9.2,11.2)	26.2	(16.6,37.0)	2.8	(0.8,6.0)
Southwest China	13.1	(10.6,15.9)	16.7	(6.5,30.5)	2.0	(0.4,4.8)
Multi-regions	10.0	(6.8,13.9)	46.2	(25.0,68.2)	6.8	(3.8,10.5)

*HBV* hepatitis B, *HCV* hepatitis C, *CI* confidence intervals

### Meta-analysis of anti-HCV prevalence in PLWHA

**Fig. 3:**
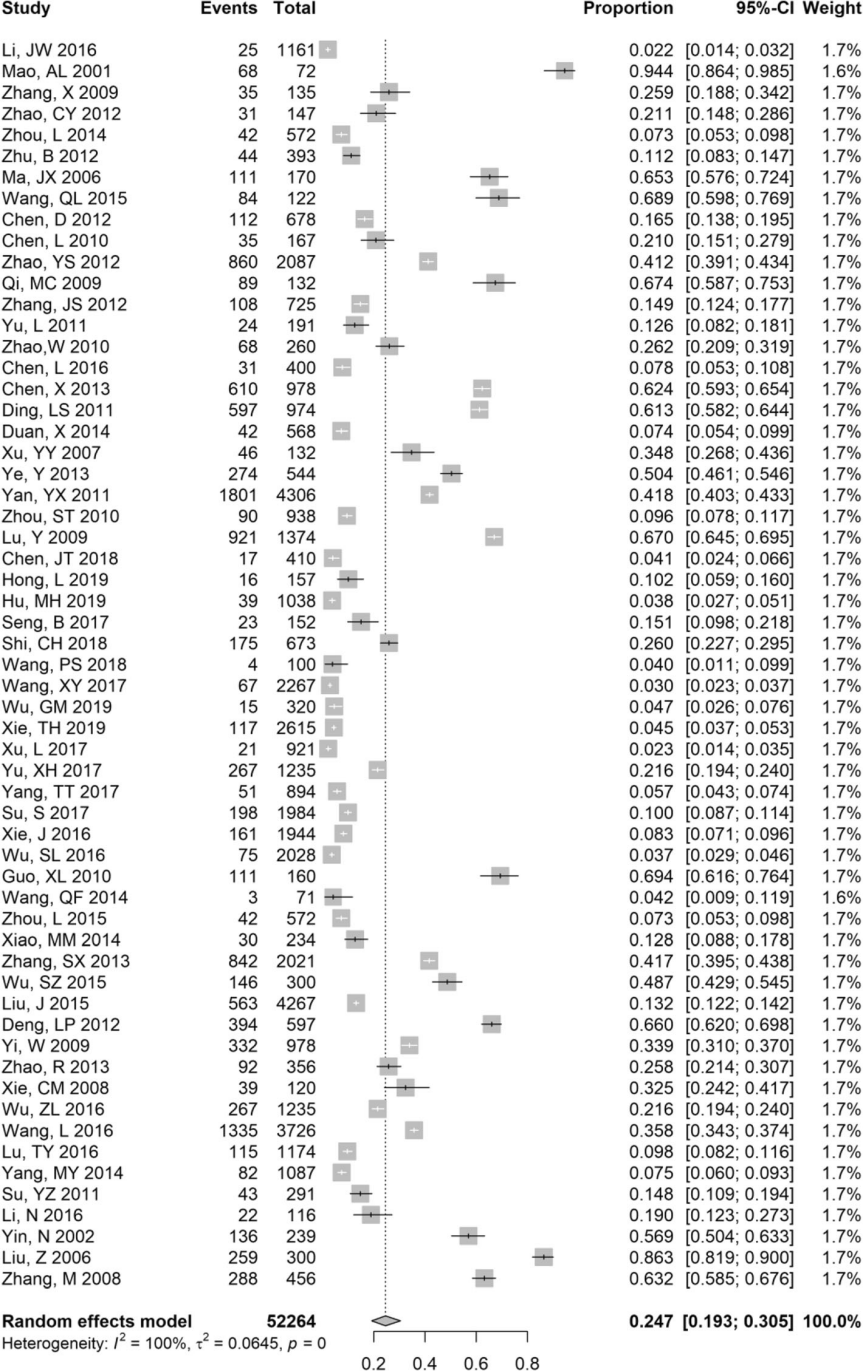
Forest plot showing meta-analysis of anti-HCV prevalence in HIV-positive populations using random effects model. The vertical dotted line indicates the pooled prevalence of all studies combined

The anti-HCV prevalence estimates (Supplementary Table 1) ranged from 2.2% (95% CI: 1.4–3.2%) to 94.4% (95% CI: 86.4–98.5%) among PLWHA. A total of 59 studies involving 52,264 PLWHA contained anti-HCV prevalence data. The meta-anti-HCV prevalence was 24.7% (95% CI 19.3–30.5%) among PLWHA (Fig. 3), and the sensitivity analyses yielded consistent results, ranging from 24 to 25% (Supplementary Fig. 2). Our analysis indicated that studies conducted before 2010 had a substantially higher anti-HCV prevalence of 31.7% (95% CI 24.8–39.2%) than the 8.5% (95% CI 5.4–12.3%) found in studies conducted after 2010. The prevalence of anti-HCV was lower in studies with a mean age above 40 (12.2, 95% CI: 3.5–25.2%) than in studies with a mean age ≤ 40 (31.1, 95% CI: 21.9–41.1%). There was also substantial variation between different geographic regions. North China (28.8, 95% CI: 12–49.5%), South China (26.3, 95% CI: 11.1–45.0%), and Northwest China (26.2, 95% CI: 16.6–37.0%) generally had higher anti-HCV prevalence in PLWHA than other regions of China (Table 1).

### Meta-analysis of HBV-HCV in PLWHA

**Fig. 4:**
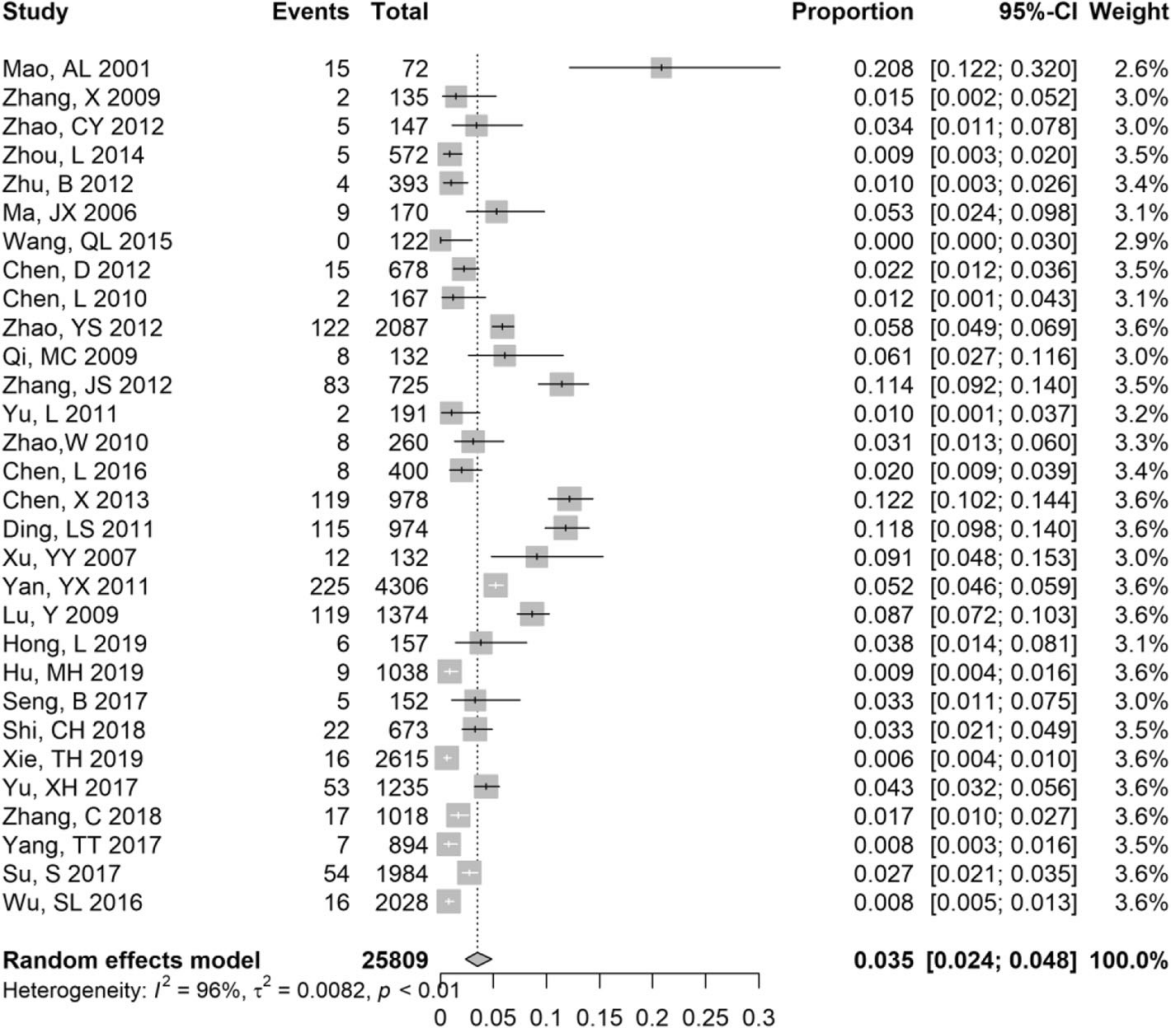
Forest plot showing meta-analysis of HBV-HCV prevalence in HIV-positive populations using random effects model. The vertical dotted line indicates the pooled prevalence of all studies combined

We estimated the HBV-HCV coinfection prevalence among 25,809 PLWHA derived from 30 studies. The pooled HBV-HCV coinfection prevalence was 3.5% (95% CI 2.4–4.8%) among PLWHA, with the lowest prevalence of 1.0% (95% CI: 0.1–3.7%) and the highest prevalence of 20.8% (95% CI: 12.2–32.0%) (Fig. 4). However, no single study was found to be highly influential on the pooled prevalence estimate in the sensitivity analysis (Supplementary Fig. 3). There was no substantial variation in the subgroup analysis, including study period, study design, proportion of gender, and study quality (Table 1). The prevalence of HBV-HCV was lower in studies with a mean age above 40 (1.5, 95% CI: 0.7–2.6%) than in studies with a mean age ≤ 40 (4.1,95% CI: 2.4–6.3%). The prevalence of HBV-HCV was generally higher in South China (4.8, 95% CI: 1.0–11.0%) and Central China (3.9,95% CI: 1.0–8.5%) than in other regions.

### Assessment of publication bias

Both Egger’s test and Peter’s test were used to explore the possible publication bias of our meta-analysis. According to the results of Egger’s test, there was no evidence of publication bias for HBV (*p* = 0.18032) and HCV (*p* = 0.43453). The results of Peter’s test indicated that there is possible publication bias for HBV (*p* = 0.04144) and no evidence of publication bias for HCV (*p* = 0.4915). However, the adjustment using the trim and fill method had no significant effect on the summary estimate for HBV (unadjusted prevalence: 13.7% vs adjusted prevalence by 13.2%).

## Discussion

People with HIV in China have a very high burden of HBV, HCV, and HBV-HCV infection, and the pooled prevalence rates of HBsAg, anti-HCV, and HBV-HCV were estimated to be 13.7, 24.7 and 3.5%, respectively, which are substantially higher than the prevalence rates in the general population. Notably, there were variations in coinfection across different subgroups. Taken together, our findings underpin the routine testing for HBV and HCV testing in all HIV-infected people. To our limited knowledge, this is the first systematic review and meta-analysis of the prevalence estimates of HBV, HCV and HBV-HCV infection in PLWHA in China. This study could help estimate the public health burden of HBV or HCV among PLWHA in China, and the findings of this study could inform further intervention design and policymaking.

China is an endemic area for hepatitis B, and it contains the greatest number of chronic HBV infections across the globe. According to the most recent national serological survey, the weighted prevalence of HBsAg in the general population was 7.2% in 2006 [27]. The pooled prevalence of HBsAg in HIV-infected individuals in our review was 13.7% (95% CI 12.3–15.3%), which is almost twice the prevalence in the general population of China. Similar findings were widely reported in other parts of the world. In Western and Central European countries, 4.9% of HIV-positive patients were HBsAg positive [28], which is substantially higher than < 1% in the general population. In Canadian HIV-positive patients, 10.46% were also coinfected with HBV [29]. However, HBsAg seroprevalence rates were estimated to be between 0.24 and 0.47% in the general Canadian population. Historically, the endemic HBV in China was largely attributed to mother-to-infant transmission as well as unsafe blood transfusion or use of contaminated blood products [27]. Our findings corroborate previously published evidence that HIV-positive people have a higher risk of having chronic HBV infection, which might be due to excess risk behaviors (i.e., unprotected sexual behavior or injection drug use) or reduced ability to clear acute HBV infection [30]. Therefore, HIV patients, even those who are HBV-seronegative, should be offered vaccination or boost vaccination to ensure that most patients can benefit.

The overall anti-HCV antibody prevalence in HIV-positive people obtained in our meta-analysis was substantially higher than the estimates from the general population in China. Globally, the high HCV prevalence in HIV-infected individuals is largely driven by injection drug use (IDU) and sexual transmission in men who have sex with men (MSM). Although our meta-analysis is not able to distinguish the driving force of high HCV infection among HIV-positive people, we speculated that this high burden of HCV infection was predominately attributed to IDU. For example, a recent study indicated a national HCV prevalence of 59.9% (95% CI: 52.7–66.7%) among methadone maintenance treatment (MMT) clients in China [31], which is consistent with the results of our meta-analysis. However, our meta-analysis also indicated that there was a sharp decrease in HCV prevalence in HIV-positive people from 31.7% (95% CI: 24.8–39.2%) in studies conducted before 2010 to 8.5% (95% CI: 5.4–12.3%) in studies conducted after 2010. This decrease could be the result of widely MMT and needle and syringe programs (NSP), which are both empirically validated interventions to curb HCV in IDUs. Despite the emergence of HCV infection epidemics in HIV-infected MSM in Western developed countries [32], there is currently no study suggesting an increase in sexual transmission of HCV in HIV-infected MSM in China to date. With the new oral DAAs available, HCV now becomes a curable disease, and the reported SVR rate in HIV-positive patients is as high as 90% [33]. Mathematical modeling predicts that if the required scale-up in DAA treatment uptake is achieved, then there should be expected substantial reductions in HCV prevalence in HIV-infected patients within a decade [34]. While cost is likely to be high, newer oral DAAs may represent the best way to treat HCV in HIV-positive people.

HIV coinfected with HBV and HCV increases morbidity and mortality beyond those caused by either infection alone [35]. People with triple infection had significantly worse virological responses than those with HIV only or HIV coinfected with only one of them [36]. Treatment options for triple infection involving HBV, HCV and HIV are also very limited, and there is currently no optimal treatment for this. In addition, people who have triple infections clearly carry a higher risk of spreading those infections to others through risk behaviors [37]. It is desirable that clinicians know the HBV/HCV status of PLWHA to understand clinical problems and choose treatment regimens. More importantly, such information can guide targeted prevention to prevent further spread of these chronic viral infections. Given the high burden of triple infections, routine screening of the high-risk population is warranted in China.

Several limitations must be admitted before interpreting our findings. First, significant heterogeneity was identified in this meta-analysis, which could not be completely explained by the stratified meta-analyses. Therefore, each prevalence estimate should be interpreted as an average prevalence across studies with true differences in target population prevalence, not a common prevalence across studies with the same target population prevalence. Second, although an extensive literature search was performed in multiple databases, it is still possible that eligible studies were missed in our search. Moreover, although we attempted to extract sufficient information from the identified literature, a large number of potentially relevant studies were identified through our systematic review, but not all data were available for extraction. Consequently, there is a risk that we missed some eligible data. Last but not least, most studies were retrospective studies with no probability sampling, and therefore, the prevalence estimates were not representative of all HIV-infected people in China. We should be cautious in generalizing our findings to other HIV-infected people, especially those from communities.

## Conclusions

To our knowledge, this study is the first systematic meta-analysis to estimate HBV and HCV prevalence in PLWHA in China. Our data demonstrate that there is a higher prevalence of HBV and HCV coinfection in PLWHA of China than in the general population. The findings of this review have implications for public health practice and policymakers. Given the high burden of HBV and HCV coinfection in HIV-positive patients, a comprehensive screening, treatment, prevention service, and vaccination incorporated into general HIV management in China is imperative.

## Supplementary information

**Additional file 1.**

## Data Availability

The datasets supporting the conclusions of this article is included within the article and its additional files.

## Abbreviations

PLWHA: People living with HIV/AIDS
ART: Antiretroviral therapy
CDC: Center for Disease Control and Prevention
HBV: Hepatitis B
HCV: Hepatitis C
