# Supplementary material for: Hepatitis B and hepatitis C prevalence among people living with HIV/AIDS in China: a systematic review and Meta-analysis
Source: Virol J. 2020 Aug 24;17:127. doi: 10.1186/s12985-020-01404-z (PMC7446115; doi:10.1186/s12985-020-01404-z)
Supplement: Supplementary file 1 — Additional file 1. [file 12985_2020_1404_MOESM1_ESM.docx]

**Supplementary**

| **Supplementary Table 1 Study Characteristics of Included 66 Studies** | | | | | | | | | | | |
| --- | --- | --- | --- | --- | --- | --- | --- | --- | --- | --- | --- |
| **Author[reference]** | **Region** | **HBV** | **HIV positive^a^** | **HCV** | **HIV positive^b^** | **Study Period** | **Population** | **Male,%** | **Age** | **Study design** | **Quality^c^** |
| Li, JW 2016[1] | North China | 69 | 1164 | 25 | 1161 | 2010~2019 | Hospital-based | 95.5 | 33.4 | Retrospective | High |
| Tian, YR 2016[2] | North China | 14 | 183 | / | / | Before 2010 | Community-based | 100.0 | 30.0 | Cross-sectional | Moderate |
| Mao, AL 2001[3] | North China | 19 | 72 | 68 | 72 | Before 2010 | Hospital-based | 95.8 | 33.3 | Retrospective | Moderate |
| Zhang,X 2009[4] | North China | 7 | 135 | 35 | 135 | Before 2010 | Hospital-based | 44.4 | 40.0 | Retrospective | Moderate |
| Zhao, CY 2012[5] | North China | 27 | 147 | 31 | 147 | Before 2010 | Community-based | 74.1 | / | Cross-sectional | Moderate |
| Zhou, L 2014[6] | East China | 91 | 572 | 42 | 572 | Before 2010 | Community-based | 72.6 | 36.0 | Retrospective | High |
| Zhu, B 2012[7] | East China | 68 | 393 | 44 | 393 | Before 2010 | Community-based | / | / | Retrospective | Moderate |
| Ma, JX 2006[8] | East China | 19 | 170 | 111 | 170 | Before 2010 | Hospital-based | 85.9 | 41.0 | Retrospective | Moderate |
| Wang, QL 2015[9] | East China | 3 | 122 | 84 | 122 | Before 2010 | Community-based | 50.0 | 37.0 | Retrospective | Moderate |
| Chen, D 2012[10] | East China | 148 | 678 | 112 | 678 | Before 2010 | Community-based | 70.4 | / | Retrospective | High |
| Chen, L 2010[11] | East China | 12 | 167 | 35 | 167 | / | Community-based | 67.7 | 40.0 | Retrospective | Moderate |
| Zhao, YS 2012[12] | East China | 262 | 2087 | 860 | 2087 | Before 2010 | Community-based | 62.8 | 31.4 | Retrospective | High |
| Qi, MC 2009[13] | South China | 26 | 132 | 89 | 132 | Before 2010 | Hospital-based | 61.3 | 32.0 | Retrospective | Moderate |
| Tang, YB 2003[14] | South China | 9 | 55 | / | / | Before 2010 | Hospital-based | 69.1 | 35.6 | Retrospective | Moderate |
| Zhang, JS 2012[15] | South China | 186 | 725 | 108 | 725 | 2010~2019 | Hospital-based | 70.1 | 39.7 | Retrospective | High |
| Feng, D 2017[16] | South China | 113 | 816 | / | / | 2010~2019 | Hospital-based | 65.1 | 37.0 | Cross-sectional | High |
| Huang,SM 2015[17] | South China | 165 | 838 | / | / | 2010~2019 | Hospital-based | 60.0 | 38.0 | Retrospective | High |
| Yu, L 2011[18] | South China | 18 | 191 | 24 | 191 | Before 2010 | Hospital-based | 0.0 | 26.0 | Retrospective | Moderate |
| Zhao,W 2010[19] | South China | 37 | 260 | 68 | 260 | / | / | / | / | Cross-sectional | Moderate |
| Chen, L 2016[20] | Central China | 119 | 400 | 31 | 400 | Before 2010 | Community-based | 68.0 | 35.0 | Retrospective | High |
| Chen, X 2013[21] | Central China | 189 | 978 | 610 | 978 | Before 2010 | Community-based | 78.0 | 39.0 | Retrospective | High |
| Ding, LS 2011[22] | Central China | 185 | 974 | 597 | 974 | Before 2010 | Community-based | 77.6 | 37.5 | Retrospective | High |
| Duan, X 2014[23] | Central China | 117 | 730 | 42 | 568 | Before 2010 | Community-based | 71.4 | 42.7 | Retrospective | High |
| Yin, T 2016[24] | Northwest China | 122 | 1235 | / | / | Before 2010 | Community-based | 85.1 | / | Retrospective | High |
| Xu, YY 2007[25] | Southwest China | 26 | 132 | 46 | 132 | Before 2010 | Hospital-based | 80.3 | 32.0 | Retrospective | Moderate |
| Ye, Y 2013[26] | Southwest China | 46 | 544 | 274 | 544 | Before 2010 | Community-based | 65.6 | 29.5 | Retrospective | High |
| Yan, YX 2011[27] | Multi-regions | 538 | 4306 | 1801 | 4306 | Before 2010 | Hospital-based | 80.6 | 36.0 | Cross-sectional | High |
| Zhou, ST 2010[28] | Multi-regions | 53 | 1082 | 90 | 938 | Before 2010 | Hospital-based | 59.7 | 7.0 | Retrospective | High |
| Lu, Y 2009[29] | Multi-regions | 197 | 1374 | 921 | 1374 | / | Community-based | / | / | Cross-sectional | High |
| Chen, JT 2018[30] | South China | 62 | 410 | 17 | 410 | 2010~2019 | Community-based | 58.3 | 46.0 | Cross-sectional | High |
| Hong, L 2019[31] | East China | 46 | 147 | 16 | 157 | 2010~2019 | Community-based | 100.0 | 41.1 | Cross-sectional | Moderate |
| Hu, MH 2019[32] | Central China | 176 | 1038 | 39 | 1038 | Before 2010 | Community-based | 83.1 | 44.1 | Retrospective | High |
| Seng, B 2017[33] | North China | 44 | 152 | 23 | 152 | Before 2010 | Hospital-based | 81.0 | / | Retrospective | Moderate |
| Shi, CH 2018[34] | North China | 122 | 673 | 175 | 673 | 2010~2019 | Community-based | 77.3 | 5-78 | Retrospective | High |
| Wang, PS 2018[35] | East China | 12 | 100 | 4 | 100 | 2010~2019 | Hospital-based | 79.0 | 45.3 | Retrospective | Moderate |
| Wang, XY 2017[36] | Southwest China | 326 | 2305 | 67 | 2267 | 2010~2019 | Hospital-based | 0.0 | / | Retrospective | High |
| Wu, GM 2019[37] | Central China | 25 | 326 | 15 | 320 | Before 2010 | Hospital-based | 0.0 | 25.0 | Retrospective | High |
| Xie, NH 2019[38] | Central China | 251 | 2615 | 117 | 2615 | Before 2010 | Community-based | 90.2 | 37.1 | Retrospective | High |
| Xu, L 2017[39] | North China | 61 | 931 | 21 | 921 | 2010~2019 | Hospital-based | 96.1 | 29.0 | Retrospective | High |
| Yang, RG 2019[40] | Southwest China | 54 | 446 | / | / | 2010~2019 | Hospital-based | 80.1 | / | Retrospective | High |
| Yu, XH 2017[41] | Northwest China | 122 | 1235 | 267 | 1235 | Before 2010 | Hospital-based | 85.1 | / | Retrospective | High |
| Zhang, C 2018[42] | Northwest China | 112 | 1018 | / | / | 2010~2019 | Hospital-based | 82.0 | 36.0 | Retrospective | High |
| Yang, TT 2017[43] | Southwest China | 129 | 894 | 51 | 894 | 2010~2019 | Hospital-based | 74.7 | 44.0 | Retrospective | High |
| Su, S 2017[44] | Central China | 184 | 1984 | 198 | 1984 | Before 2010 | Community-based | 66.7 | 39.0 | Retrospective | High |
| Xie, J 2016[45] | Multi-regions | 186 | 1944 | 161 | 1944 | Before 2010 | Community-based | 67.0 | 36.0 | Cross-sectional | High |
| Wu, SL 2016[46] | East China | 329 | 2028 | 75 | 2028 | / | Community-based | 72.5 | 40.8 | Retrospective | High |
| Guo, XL 2010[47] | North China | / | / | 111 | 160 | Before 2010 | Community-based | 74.3 | / | Cross-sectional | Low |
| Wang, QF 2014[48] | East China | / | / | 3 | 71 | Before 2010 | Community-based | 88.7 | / | Retrospective | Moderate |
| Zhou, L 2015[49] | East China | / | / | 42 | 572 | Before 2010 | Community-based | 72.6 | 38.5 | Retrospective | High |
| Xiao, MM 2014[50] | East China | / | / | 30 | 234 | 2010~2019 | Community-based | 65.4 | / | Retrospective | High |
| Zhang, SX 2013[51] | East China | / | / | 842 | 2021 | Before 2010 | Community-based | 62.7 | 35.0 | Retrospective | High |
| Wu, SZ 2015[52] | South China | / | / | 146 | 300 | Before 2010 | Community-based | 65.0 | 37.9 | Retrospective | High |
| Liu, J 2015[53] | Central China | / | / | 563 | 4267 | 2010~2019 | Community-based | 73.9 | / | Retrospective | High |
| Deng, LP 2012[54] | Central China | / | / | 394 | 597 | Before 2010 | Hospital-based | / | / | Retrospective | Moderate |
| Yi, W 2009[55] | Central China | / | / | 332 | 978 | Before 2010 | Hospital-based | 69.9 | / | Retrospective | High |
| Zhao, R 2013[56] | Central China | / | / | 92 | 356 | Before 2010 | Community-based | 73.6 | / | Retrospective | Moderate |
| Xie, CM 2008[57] | Central China | / | / | 39 | 120 | / | / | / | / | Cross-sectional | Low |
| Wu, ZL 2016[58] | Northwest China | / | / | 267 | 1235 | Before 2010 | Community-based | 85.1 |  | Retrospective | High |
| Wang, L 2016[59] | Northwest China | / | / | 1335 | 3726 | Before 2010 | Community-based | 57.9 | 40.5 | Retrospective | High |
| Lu, TY 2016[60] | Central China | / | / | 115 | 1174 | 2010~2019 | Community-based | / | / | Retrospective | Moderate |
| Yang, MY 2014[61] | Southwest China | / | / | 82 | 1087 | 2010~2019 | Community-based | 72.9 | / | Retrospective | High |
| Su, YZ 2011[62] | Southwest China | / | / | 43 | 291 | / | / | / | / | Cross-sectional | Moderate |
| Li, N 2016[63] | Southwest China | / | / | 22 | 116 | 2010~2019 | Community-based | 62.0 | / | Retrospective | Low |
| Yin, N 2002[64] | Multi-regions | / | / | 136 | 239 | Before 2010 | Hospital-based | 66.1 | 33.0 | Retrospective | High |
| Liu, Z 2006[65] | Multi-regions | / | / | 259 | 300 | Before 2010 | Hospital-based | 50.0 | 39.0 | Retrospective | High |
| Zhang, M 2008[66] | Multi-regions | / | / | 288 | 456 | Before 2010 | Community-based | 58.2 | 38.8 | Cross-sectional | High |

a, The number of HIV positive patients who had HBV test; b, The number of HIV positive patients who had HCV test; c,high means quality score is ≥3, Moderate means quality score is 2, Low means quality score is 1.

HBV=Hepatitis B; HCV=Hepatitis C; HIV= Human Immunodeficiency Virus

**Supplementary Figures**


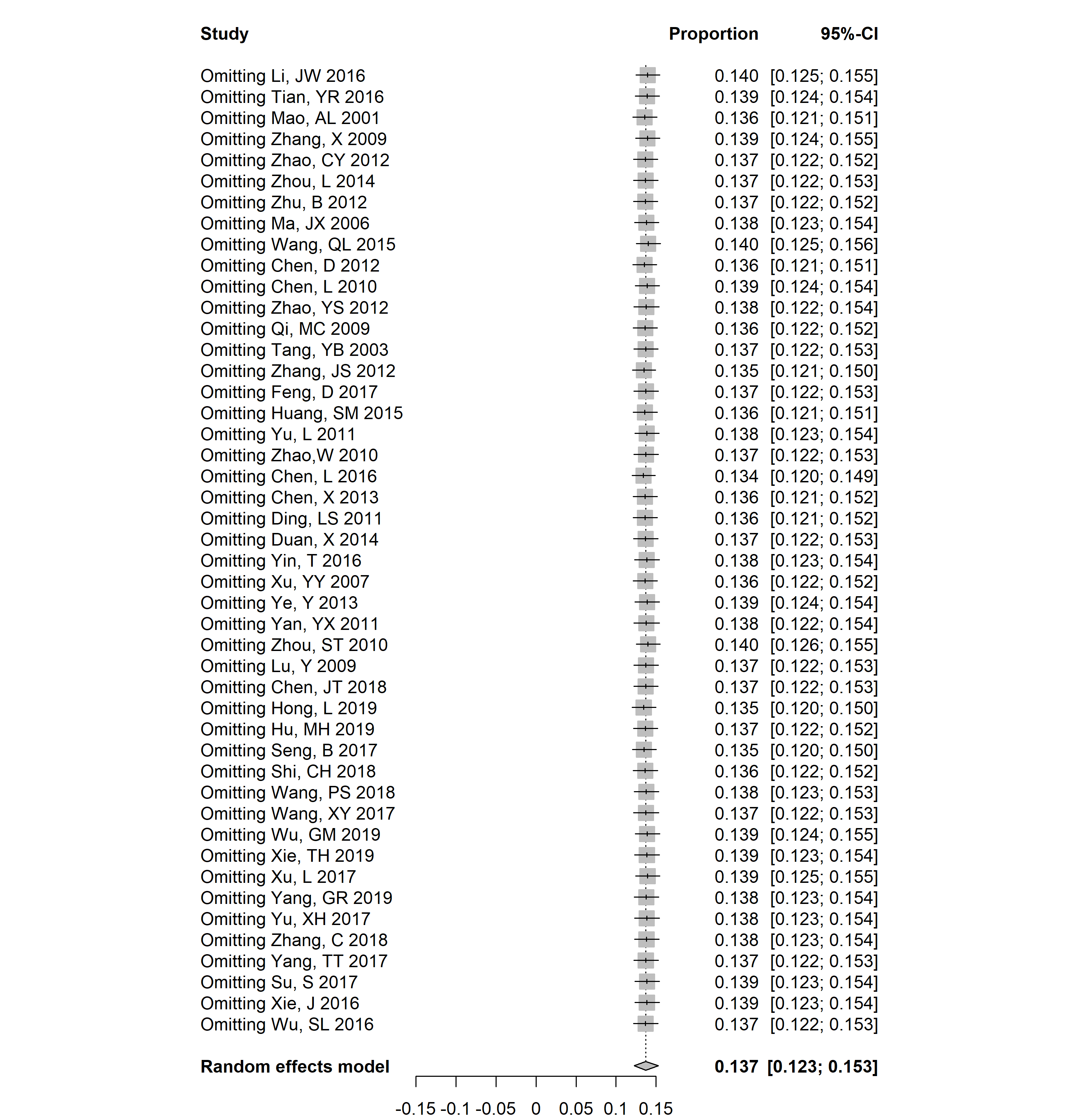


**Supplementary Figure 1. Forest plot showing influential analysis when omitting each individual study for meta-analysis of HBV prevalence.** The vertical dotted line indicates the pooled prevalence of all studies combined.


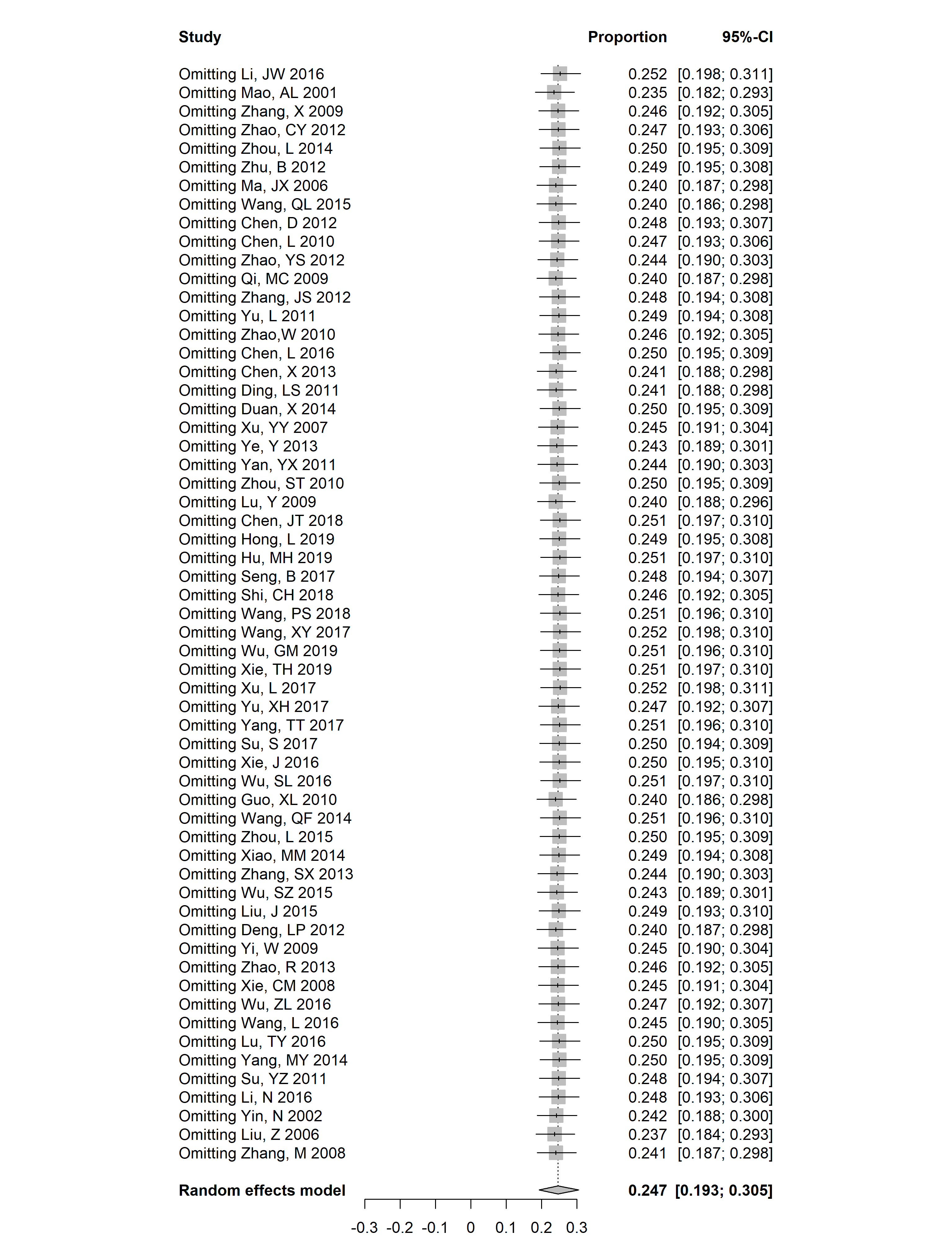


**Supplementary Figure 2. Forest plot showing influential analysis when omitting each individual study for meta-analysis of HCV prevalence.** The vertical dotted line indicates the pooled prevalence of all studies combined.


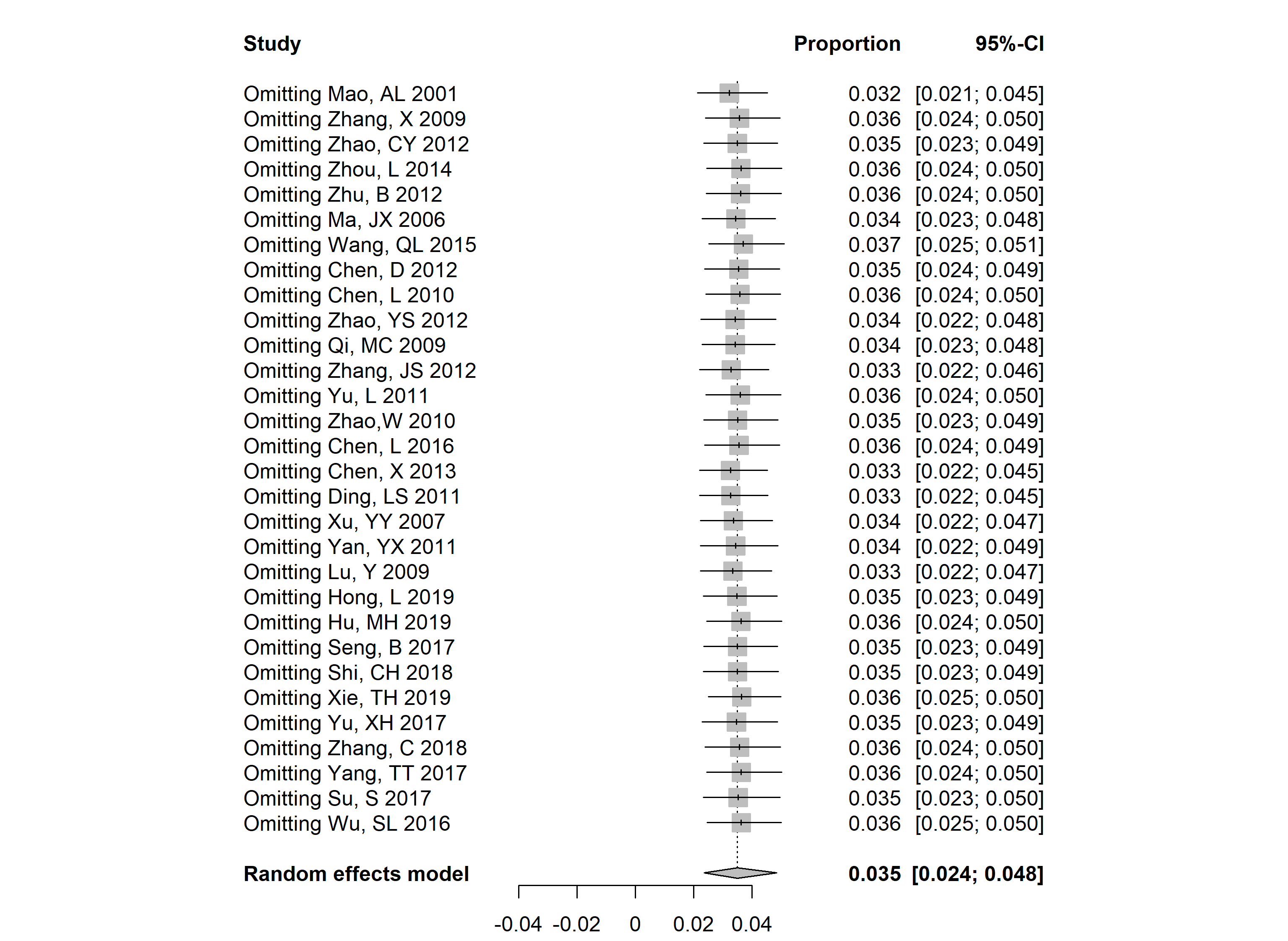


**Supplementary Figure 3. Forest plot showing influential analysis when omitting each individual study for meta-analysis of HBV-HCV prevalence.** The vertical dotted line indicates the pooled prevalence of all studies combined.

**Supplementary Reference**

1. Jianwei L, Xiaofeng Z, Aiping L, Ying S, Jiangzhu Y, Liang H, Hongli M, Zaicun L: **Analysis of HBV HCV and TP infection among HIV/AIDS patients in Youan Hospital.** *Chinese Journal of AIDS & STD* 2016, **22:**608-610.

2. Yaru T: **Study on the co-infection of HBV in the progress of HIV infection among men who have sex with men.** *硕士.* Capital Medical University, 2016.

3. Mao A, Guo X, Qiao X, Nie X, Zhao Y: **Investigation of 72 HIV-infected persons complicated with HBV HCV syphilis infection.** *Preventive Medicine Tribune* 2001, **7:**692.

4. Xin Z, Huiping Y, Yufen T, Yan L, Haiping Z, Dongmei M, Yongmei X: **Investigation of the characteristics of 135 HIV/AIDS patients infected with HBV and/or HCV virus.** *Immunological Journal* 2009, **25:**362-363.

5. Cuiying Z, Qiaomin L, Hongru Z, Xinli L, Guangyi B, Yan L, Yingying W, Wei W: **Study on HCV and HBV co-infection among HIV-1 infected people in Hebei Province.** *Chinese Journal of AIDS & STD* 2012, **18:**521-523.

6. Lin Z, Qionghai W, Weiwei S, Yingying D, Haijiang L, Na H: **HBV Coinfection among Newly Diagnosed HIV-infected Adults in Taizhou of Zhejiang.** *Chinese Primary Health Care* 2014, **28:**71-74.

7. Bing Z, Dong C, Baochang S, Chengchao Y: **Research on effect of AIDS coinfection with HBV, HCV and TP on CD4.** *Chinese Journal of Health Laboratory Technology* 2012, **22:**2114-2115,2118.

8. Jianxin M, Jiangrong W, Yinzhong S, Renfang Z, Xinian L, Xueyan J, Hongqing S, Hongzhou L: **Clinical epidemiology studies on HIV-1/AIDS subjects co-infected with HBV and/or HCV in Shanghai.** *Journal of Microbes and Infections* 2006, **1:**207-210.

9. Qiliang W, Minghua L, Zongli W: **Clinical epidemiology of HIV/AIDS subjects co-infection in patients with HCV and/or HBV.** *Anhui Medical Journal* 2015, **36:**583-586.

10. Dong C, Lili C, Huifen D, Chengchao Y, Yuejin W, Airong X: **Research on the situation of HIV/AIDS coinfected with HBV, HCV, TP and other diseases in Wenzhou area.** *Chinese Journal of Health Laboratory Technology* 2012, **22:**587-590.

11. Li C, Haijiang L, Jifu F, Danhong Q, Meiyang G, Na H: **HBV, HCV and EBV coinfection among HIV carriers in Taizhou city, China.** *China Preventive Medicine* 2010, **11:**442-445.

12. Yusui Z: **HCV, HBV and TP Infection of HIV/AIDS Patients in Shandong Province and Related Research.** *硕士.* University of Jinan, 2012.

13. Maochao Q, Shili L: **Influences of sharing needles on the infection rates of hepatitis C, hepatitis B and syphilis in HIV-infected persons.** *Chinese Journal of Misdiagnostics* 2009, **9:**7633-7633.

14. Yangbo T, Xiejie C, Xiaoping T, Weilie C, Xiaozhen Y: **Analysis on Clinical and Epidemic Features of HIV Infectors in Guangzhou.** *Chinese Journal of Clinical Medicine* 2003, **10:**446-447.

15. Jiansheng Z, Jingmin N, Haolan H, Weiping C: **Epidemiological investigation on HIV/AIDS patients in Guangdong Province in 2011.** *Infectious Disease Information* 2012, **25:**356-359.

16. Dan F, Tian Y, Yanpeng C, Minghu P, Chunxia L, Jun W, Yongliang F, Jing S, Honglang H, Hongyan L, et al: **Prevalence and related factors of HIV/HBV coinfection among HIV/AIDS patients.** *Chinese Journal of Epidemiology* 2017, **38:**1624-1628.

17. Simin H, Weiping C, Fengyu H, Baolin L, Yun L, Youpeng C, Xiaoping T: **Epidemiological and clinical features of human immunodeficiency virus/hepatitis B virus co-infected patients.** *Chinese Journal of Infectious Diseases* 2015**:**391-395.

18. Lan Y, Xiuzhi G, Xiuning M, Ziqi B, Hongyan L, Wenying L, Fujie Z: **Incidence of HBV, HCV and syphilis co-infections among 194 HIV-infected pregnant women.** *Chinese Journal of AIDS & STD* 2011, **17:**308-310.

19. Wen Z, Xi'an Y, Yangbo T, Hanlin Z: **Analysis of HBV and HCV infection in HIV-infected persons.** *Guangdong Medical Journal* 2010, **31:**79-81.

20. Li C, Xiaoyan Y, Fang W: **Investigation and Analysis of Infection of Hepatitis B, Hepatitis C and Syphilis in HIV Infected Persons.** *The Medical Forum* 2016, **20:**3105-3106.

21. Chen X, He J-M, Ding L-S, Zhang G-Q, Zou X-B, Zheng J: **Prevalence of hepatitis B virus and hepatitis C virus in patients with human immunodeficiency virus infection in central China.** *Archives of virology* 2013, **158:**1889-1894.

22. Lisha D, Guoqiang Z, Yang J, Biyun Q, Jianmei H, Xi C, Xiaobai Z, Fujie Z: **Infection status and risk factors of HBV and HCV among HIV-infected people in Hunan,China.** *Chinese Journal of Viral Diseases* 2011, **1:**358-362.

23. Xiang D: **Survival status and influencing factors of 2103 AIDS patients in Jiangxi Province.** *硕士.* Nanchang University, 2014.

24. Ting Y, Zhonglan W, Lihua Z, Dongzhi Y, Min C, Huizhong W: **Analysis of HIV/AIDS and HBV infection in Ningxia.** *Journal of Ningxia Medical University* 2016, **38:**408-411.

25. Yunya X, Song H, Xinhua W, Ming L, Yan M, Hui L, Lilin Z: **The clinical analysis on 132 AIDS cases of opportunistic infections.** *Soft Science of Health* 2007, **21:**513-516.

26. Yong Y, Hongmei S, Aikun Y, Yunde L: **Analysis of detection results of HIV infection with HBV, HCV and HGV in Puer City.** *Soft Science of Health* 2013**:**581-584.

27. Yan YX, Gao YQ, Sun X, Wang W, Huang XJ, Zhang T, Li M, Zang CP, Li ZC, Wu H: **Prevalence of hepatitis C virus and hepatitis B virus infections in HIV-positive Chinese patients.** *Epidemiol Infect* 2011, **139:**354-360.

28. Zhou S, Zhao Y, He Y, Li H, Bulterys M, Sun X, Dou Z, Robinson M, Zhang F: **Hepatitis B and hepatitis C seroprevalence in children receiving antiretroviral therapy for human immunodeficiency virus-1 infection in China, 2005-2009.** *J Acquir Immune Defic Syndr* 2010, **54:**191-196.

29. Ying L: **Study on the prevalence of HBV and HCV among HIV-infected people in some areas of central China.** *硕士.* Lanzhou University, 2009.

30. Jiatu C, Mingqing X, Caihua Z, Guodong L, Xuemei L, Shuping P: **Situtation of HIV patients co-infected with TP, HBV and HCV in Maoming city and its relationship with CD4+ T lymphocytes.** *Chinese Journal of AIDS & STD* 2018, **24:**977-980.

31. Liang H, Jing T, Jing Z, Yiling T: **Cross-sectional Study on the Co-infection of Hepatitis B and Hepatitis C Virus among HIV-infected People in Shanghai.** *Medical Information* 2019, **32:**145-147.

32. Maohong H, Huabao S, Changhua F, Ta H, Liang L, Dan X, Xianfeng Z: **Characterization of HBV or HCV co-infection in HIV infections/AIDS patients in Jiangxi Province.** *Occupation and Health* 2019, **35:**213-217.

33. Bo S, Meng X, Lan L, Zixuan Y, Yunxia J, Rui W, Hao W, Hongquan C: **Clinical analysis of HIV/AIDS patients with hepatitis B, hepatitis C virus infection and blood indicators.** *Chinese Journal of Experimental and Clinical Infectious Diseases(Electronic Version)* 2017, **11:**377-381.

34. chaohui S: **Analysis of new AIDS epidemic and syphilis infection with hepatitis B and C in Pingdingshan City.** *Shanxi Medical Journal* 2018, **47:**997-998.

35. Peisheng W, Ruixia L, Li L: **Study on the Infection Status of Hepatitis B, Hepatitis C, Hepatitis E and Treponema pallidum in 100 HIV Infected Persons.** *Chinese Journal of Convalescent Medicine* 2018, **27:**558-560.

36. Xueyan W, Zhenkai Z, Xingqiang W, Qinyan C, Yun Z, Qingli Y, Chao W, Kaiwen L, Liping H: **The epidemiology of co-infection with HBV and/or HCV among individuals who were newly recruited for anti-HIV therapy in two cities of Guangxi between 2014 and 2015.** *Journal of Applied Preventive Medicine* 2017, **23:**439-444.

37. Guomin W, Xi'en G, Jiabing L, Jiarong L, Guizhen T, Yingyu P, Rong L, Zheng L, Tao W, Ke L: **Analysis on epidemiological characteristics and termination of pregnancy in 520 HIV-positive pregnant women in Hubei from 2004 to 2016.** *Maternal & Child Health Care of China* 2019, **34:**405-410.

38. Nianhua X, Xia W, Si W, Han Y, Wang Z: **Epidemiological Characteristics and Influencing Factors of HBV and HCV Coinfections among Patients with HIV/AIDS in Wuhan City.** *Acta Medicinae Universitatis Scientiae et Technologiae Huazhong* 2019, **48:**183-188.

39. Lin X, Guorui D, Xuejing Z, Di W, Jiang X, Guiju G, Di Y, Hongxin Z: **Prevalence of HIV/HBV, HIV/HCV and HIV/syphilis co-infections.** *Chinese Journal of AIDS & STD* 2017, **23:**138-141.

40. Renguo Y, Kaiju X, Xingxiang Y: **Analysis of HIV/AIDS patients complicated with HBV infection in a third-class A hospital.** *Journal of Military Surgeon in in Southwest China* 2019, **21:**126-130.

41. Xiaohong Y, Zhonglan W, Guangyu G, Ting Y, Dongzhi Y, Min C, Xuemin M: **Analysis of HIV/AIDS Complicated with Hepatitis B, Hepatitis C and Syphilis in Ningxia.** *Journal of Ningxia Medical University* 2017, **39:**1426-1429.

42. Chao Z, Hua L, Qiang R, Yangfan Z, Yulin F, Mengyan Z, Wenhui C: **Analysis of 1018 HIV/AIDS patients co-infected with HBV, HCV, and TP in Shaanxi.** *China Tropical Medicine* 2018, **18:**999-1003.

43. Yang T, Chen Q, Li D, Wang T, Gou Y, Wei B, Tao C: **High prevalence of syphilis, HBV, and HCV co-infection, and low rate of effective vaccination against hepatitis B in HIV-infected patients in West China hospital.** *J Med Virol* 2018, **90:**101-108.

44. Su S, Fairley CK, Sasadeusz J, He J, Wei X, Zeng H, Jing J, Mao L, Chen X, Zhang L: **HBV, HCV, and HBV/HCV co-infection among HIV-positive patients in Hunan province, China: Regimen selection, hepatotoxicity, and antiretroviral therapy outcome.** *J Med Virol* 2018, **90:**518-525.

45. Xie J, Han Y, Qiu Z, Li Y, Li Y, Song X, Wang H, Thio CL, Li T: **Prevalence of hepatitis B and C viruses in HIV-positive patients in China: a cross-sectional study.** *J Int AIDS Soc* 2016, **19:**20659.

46. Wu S, Yan P, Yang T, Wang Z, Yan Y: **Epidemiological profile and risk factors of HIV and HBV/HCV co-infection in Fujian Province, southeastern China.** *J Med Virol* 2017, **89:**443-449.

47. Xiaoli G, Naichang W, Bo Z: **Preliminary Analysis of the HCV Infection among Certain Population in Shanxi Province.** *Chinese Journal of Public Health Management* 2010, **26:**288-289.

48. Qingfeng W, Qinghua G, Meifeng S: **Analysis of HCV and TP in HIV-infected persons in Changshu City.** *Jiangsu Journal of Preventive Medicine* 2014, **25:**63-64.

49. Lin Z, Qionghai W, Weiwei S, Meiyang G, Yingying D, Haijiang L, Na H: **Co-infection of hepatitis C virus among newly diagnosed HIV-infected adults in Taizhou prefecture of Zhejiang province, China.** *Chinese Journal of Epidemiology* 2015, **36:**862-866.

50. Minmin X, Yi W, Hui S, Yan Z, Ping L: **Investigation of HCV co-infection among HIV-1 infected patients in the South of Anhui.** *Laboratory Medicine* 2014**:**705-707.

51. Shuxia Z, Shengli S, Bin L, Xiaoguang S, Jihua F: **Epidemiological analysis of HIV and HCV co-infection in Shandong.** *Chinese Journal of AIDS & STD* 2013, **19:**110-113.

52. Shuzhi W, Jinlu W, Zhichun X: **Detection and analysis of HIV/HCV co-infection in Guangxi.** *China Journal of Modern Medicine* 2015, **25:**30-34.

53. Jia L, Panying F, Xiujuan X, Guoqing S, Chunhua L, Sui'an T, Jie L, Ning L, Dingyong S: **Prevalence of hepatitis C virus antibody among newly reported HIV infection cases in Henan,2012-2014.** *Chinese Journal of Epidemiology* 2015, **36:**1269-1273.

54. Liping D, Xi'en G, Shicheng G, Yong X, Rongrong Y, Mingqi L: **Epidemiology and distribution of hepatitis C virus genotype among HIV positive former blood donors and transfusion recipients in Hubei province.** *Chinese Journal of General Practitioners* 2012, **11:**441-443.

55. Wei Y: **Retrospective analysis of HCV, TB infection and HIV infection among HIV-infected patients in Hunan Province.** *硕士.* Central South University, 2009.

56. Zhao R, Peng J, Tang L, Huang H, Liu M, Kong W, Pang B: **Epidemiological distribution and genotype characterization of hepatitis C virus and HIV co-infection in Wuhan, China, where the prevalence of HIV is low.** *J Med Virol* 2013, **85:**1712-1723.

57. Chaomei X, Xipeng Z, Jing S: **Detection and analysis of blood indicators in HIV carriers.** *Chinese Journal of Health Laboratory Technology* 2008, **18:**1979-1981.

58. Zhonglan W, Weiming Z, Min C, Dongzhi Y, Guangyu G, Hui C, Jiangtao M, Xuemin M: **Prevalence and associated risk factors of HIV/HCV co-infection in Ningxia province.** *Chinese Journal of AIDS & STD* 2016, **22:**81-83.

59. Li W, Tao C, Xinmei S, Xiaoxia G, Jie Z, Wei L: **The Epidemiology and Clinical Features Among AIDS Patients Taking Highly Active Antiretroviral in Yili City, Xinjiang Uygur Autonomous Region.** *The Chinese Journal of Dermatovenereology* 2016, **30:**271-273,320.

60. Tingyi L, Mingxiang W, Yaoping W, Jinhong P: **The overlapping infection status and the related research of syphilis and hepatitis C virus with AIDS in Qiannan areas.** *Chinese Journal of Health Laboratory Technology* 2016, **26:**1319-1321.

61. Maoyu Y, Jie Y: **Analysis on Test Results of Infected HIV-1 Merger Syphilis and Hepatitis C in Northeast Area of Chongqing.** *Chinese Primary Health Care* 2014, **28:**86-88.

62. Yingzhen S, Yanling M, Manhong J, Lin L, Li Y, Ling C, Huichao C, Zhaojun Y, Yuhua S, Shouyi Y: **The analysis of immune characters and features of co-infection of HCV and HSV-2 among 291 HIV patients.** *Journal of Tropical Medicine* 2011, **11:**23-25,53.

63. Na L: **Analysis of HIV-infected persons with HCV and TP infection in Dongxing District of Neijiang City.** *China Health Care & Nutrition* 2016, **26:**68-69.

64. Ning Y, Shan M, Linqi Z, Cengquan Z, Weiquan L, Yun H, Fujie Z, Yunzhen C: **Outbreak of HIV and HCV co-infection among intravenous drug users and illegal blood donors in China.** *Chinese Journal of Infection and Chemotherapy* 2002, **2:**67-69.

65. Zhen L, Wenge X, Yonghong Z, Qi Z, Xiaoshan L, Guiyun Z, Hao W, Yan J: **Study on the epidemiology and HCV genotype distribution of HIV/HCV co-infection among HIV infected blood donors in China.** *Chinese Journal of Hepatology* 2006, **14:**464-465.

66. Min Z, Qinghai H, Fei Z, Feng S, Song D, Xiangdong M, Chunming L, Jing L, Haibo D, Xia F, Hong S: **Investigation of HIV co-infection with hepatitis C virus through different infectious routes.** *Chinese Journal of Public Health Management* 2008, **24:**1409-1411.
